# An Efficient SM9 Aggregate Signature Scheme for IoV Based on FPGA

**DOI:** 10.3390/s24186011

**Published:** 2024-09-17

**Authors:** Bolin Zhang, Bin Li, Jiaxin Zhang, Yuanxin Wei, Yunfei Yan, Heru Han, Qinglei Zhou

**Affiliations:** 1 School of Computer and Artificial Intelligence, Zhengzhou University, Zhengzhou 450001, China; zhangbolin918@gmail.com (B.Z.); jxzhang@gs.zzu.edu.cn (J.Z.); weiyuanxin_zz@citicbank.com (Y.W.); 18239697238m@sina.cn (Y.Y.); h2141235277@gs.zzu.edu.cn (H.H.); ieqlzhou@zzu.edu.cn (Q.Z.); 2 China CITIC Bank Co., Ltd., Zhengzhou Branch, Zhengzhou 450008, China

**Keywords:** SM9, FPGA, fault-tolerant, aggregate signature, Internet of Vehicles

## Abstract

With the rapid development of the Internet of Vehicles (IoV), the demand for secure and efficient signature verification is becoming increasingly urgent. To meet this need, we propose an efficient SM9 aggregate signature scheme implemented on Field-Programmable Gate Array (FPGA). The scheme includes both fault-tolerant and non-fault-tolerant aggregate signature modes, which are designed to address challenges in various network environments. We provide security proofs for these two signature verification modes based on a K-ary Computational Additive Diffie–Hellman (K-CAA) difficult problem. To handle the numerous parallelizable elliptic curve point multiplication operations required during verification, we utilize FPGA’s parallel processing capabilities to design an efficient parallel point multiplication architecture. By the Montgomery point multiplication algorithm and the Barrett modular reduction algorithm, we optimize the single-point multiplication computation unit, achieving a point multiplication speed of 70776 times per second. Finally, the overall scheme was simulated and analyzed on an FPGA platform. The experimental results and analysis indicate that under error-free conditions, the proposed non-fault-tolerant aggregate mode reduces the verification time by up to 97.1% compared to other schemes. In fault-tolerant conditions, the proposed fault-tolerant aggregate mode reduces the verification time by up to 77.2% compared to other schemes. When compared to other fault-tolerant aggregate schemes, its verification time is only 28.9% of their consumption, and even in the non-fault-tolerant aggregate mode, the verification time is reduced by at least 39.1%. Therefore, the proposed scheme demonstrates significant advantages in both error-free and fault-tolerant scenarios.

## 1. Introduction

Currently, with the rapid development of the Internet of Things (IoT), an increasing number of vehicles are becoming intelligent, giving rise to the Internet of Vehicles (IoV). Within the same regional network, different IoV components exchange information through their self-organizing networks. However, IoV faces numerous challenges, such as communication delays, resource limitations, and complex communication environments. Efficiently verifying a large number of signatures using edge computing resources, while ensuring the accuracy and security of the information, is therefore a critical challenge [1,2]. To address this problem, current methods for accelerating signature verification primarily fall into two categories: (1) Compression Techniques: These methods aim to reduce the length of information to accelerate computation, such as through aggregate signature technology. (2) Algorithm Optimization: These methods focus on hardware and software optimization of the algorithms involved in signature verification, such as optimizing elliptic curve algorithms and leveraging Field-Programmable Gate Array (FPGA) hardware acceleration. However, aggregate signatures still face several issues, such as relatively long verification times and the potential for batch signature invalidation. Techniques such as parallel point multiplication acceleration and fault-tolerant aggregate signatures can effectively address these issues.

In order to address the challenge of efficiently verifying a large number of signatures, various scholars have proposed their own schemes based on different computational hardness problems and from distinct perspectives for improvement. Xie [3] improved the CLAS aggregate signature scheme based on the Elliptic Curve Discrete Logarithm Problem (ECDLP). While this reduced the time complexity of various stages of the aggregate signature process, it did not adequately address the issues of numerous point multiplication operations and fault tolerance. An [4] modified the SM9 algorithm to support aggregate signatures, improving its performance; however, the number of point multiplication operations remains high, and fault tolerance issues were not resolved. Mei [5] designed an aggregate signature scheme aimed at addressing location privacy concerns in vehicular networks, effectively ensuring the integrity and authenticity of information, but further improvements in fault tolerance and elliptic curve acceleration remain possible. Ran [6] further enhanced the elliptic curve-based aggregate signature scheme, making it more suitable for IoT applications, yet fault tolerance was not considered. Yang [7] applied aggregate signature modifications to the Schnorr signature to adapt it to wireless communication networks in the medical IoT environment, but these modifications introduced a significant number of elliptic curve operations, leaving room for optimization in terms of efficiency. Fu [8], to solve the problem of numerous point multiplication operations in aggregate signatures, accelerated these operations using FPGA, but the fault tolerance issue was still unresolved. To tackle the fault tolerance problem in aggregate signatures, Hartung [9] proposed the concept of fault-tolerant aggregate signatures and provided theoretical derivations, but this concept has not been applied to practical schemes. Therefore, for the efficient optimization of aggregate signatures, accelerating point multiplication operations and introducing fault tolerance are crucial.

In current aggregate signature schemes, the point multiplication operation is a critical path that is relatively time consuming. There are various methods for point multiplication, such as the binary expansion method, sliding window method [10], and window non-adjacent form (w-NAF) method [11]. However, there is still room for optimization in both software and hardware implementations. To improve the efficiency of point multiplication algorithms, many scholars have conducted research from different perspectives. In software, Hai et al. [12] proposed replacing odd numbers with prime numbers during the pre-computation phase and constructing micro multi-base chains to compensate for the differences between primes and odd numbers, reducing the computational complexity of scalar multiplication by up to 77.38%. Zhao et al. [13] further optimized the computational complexity by replacing the odd numbers in the base chains of the w-NAF chain with the form of $2^{n}$. In hardware: Bellemou et al. [14] used the Montgomery power ladder binary method and an improved Radix-$2^{32}$ Montgomery modular multiplication to optimize elliptic curve point multiplication, achieving a computation time of 14.32 ms and balancing security and efficiency. Yue Hao et al. [15], based on the Zynq platform, utilized the Montgomery ladder algorithm and adaptively modified point addition and point double, optimizing the computation time to 1.80 ms. Islam et al. [16], also using the Montgomery ladder algorithm on the Virtex-7 platform, proposed a Radix-2 modular multiplication architecture on the Edwards curve, optimizing the computation time to 1.63 ms.

The SM9 [17] identity-based encryption algorithm was proposed in 2016 and officially became an international standard in 2021. Numerous scholars have researched and applied this algorithm. For instance, as mentioned earlier, the SM9 aggregate signature proposed by An Tao [4] added aggregation functionality to the SM9 signature. Additionally, Liu et al. [18] combined threshold signatures, ring signatures, and SM9 to achieve higher security but did not optimize for time consumption. Liu et al. [19] proposed a two-party cooperative signature based on SM9 and applied it to smart homes, although there remains significant room for optimization in verification efficiency. Jing et al. [20] introduced a four-stage pipeline improved modular multiplication algorithm and enhanced point addition and point double algorithms. Tested on the Zynq FPGA platform based on SM9, the point multiplication algorithm achieved a performance of 1179 operations per second, though further improvements could be made in utilizing FPGA board resources. Wang et al. [21] designed highly parallel computational unit structures and optimized $F_{p}$ and $F_{p^{2}}$ domain point addition, point double, and line functions. Based on the SM9 algorithm, they designed optimal ATE computation units on the Virtex-7 FPGA platform, reducing computation time to 3.43 ms, which is 20% of the time consumed by similar designs. Cheng [22] reduced the hardware cost of the SM9 algorithm by simplifying the Frobenius map and implemented its parallel structure. However, FPGA optimization for bilinear pairings is quite resource intensive, and optimizing point multiplication operations is crucial for improving verification efficiency in aggregate signature verification. Therefore, how to fully utilize edge resources to accelerate point multiplication and apply it to specific schemes is a critical issue.

In our work, (1) we design a comprehensive scheme for SM9 aggregate signatures based on FPGA. (2) We employ a parallel hardware structure to accelerate the computation speed of aggregate signature verification. (3) We utilize an improved Montgomery ladder algorithm to compute $F_{p}$ domain point multiplications in SM9 verification and optimize the Barrett modular reduction algorithm to better suit the characteristics of SM9. (4) We implement a fault-tolerant mechanism within the aggregate signature scheme and analyze and prove the scheme’s security based on the K-ary Computational Additive Diffie–Hellman (K-CAA) difficult problem.

The rest of the paper is organized as follows: In Section 2, we introduce the preliminary knowledge and related work pertinent to our work, mainly including the SM9 signature algorithm, aggregate signatures, K-CAA problem, and fault tolerance mechanisms. In Section 3, we describe the overall architecture of our proposed scheme In Section 4, we present the hardware design, optimization strategies employed, and the fault-tolerant mechanism design. In Section 5, we analyze the experimental results. In Section 6, we provide a conclusion and outlook for future work. 

## 2. Related Work

Our work primarily involves the SM9 signature and verification algorithm, aggregate signatures, the K-CAA problem in hardness issues, and fault tolerance mechanisms. The background and related work are introduced in four parts below.

### 2.1. SM9 Signature Algorithm

**Figure 1:**
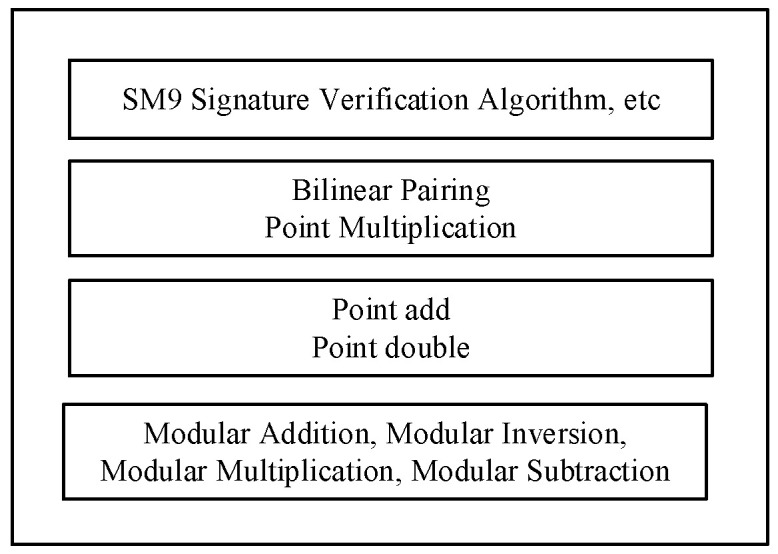
Overall architecture of SM9 algorithm.

As shown in Figure 1, the SM9 algorithm can be divided into four levels in terms of functionality and complexity. The first level is the SM9 protocol layer, which includes the signature algorithm, key exchange protocol, and others. The second level consists of bilinear pairing and point multiplication, which are more complex functional components based on elliptic curve operations. The bilinear pairing $e\left(a,b\right)$ operation rules are quite special and can be referenced in [23]. The first level achieves its functionality by invoking these components. The third level includes point addition and point double, which are the basic elliptic curve operations. The fourth level comprises modular addition, modular inversion, modular multiplication, and modular subtraction over finite fields [24].

### 2.2. Aggregate Signature

The current focus of research on encryption mechanisms is on computational efficiency and memory optimization [25]. To address these issues, many aggregate signature schemes have been developed [2,3,4,5,6,7]. Most aggregate signature algorithms involve the following steps: sign, single signature verification, aggregate signature generation, and aggregate signature verification. Below are the descriptions and explanations of these algorithms:

(a) Sign: The user, holding the signature private key $ds_{i}$ issued by the Key Generation Center (KGC), generates a signature $\sigma_{i}$ for the message $m_{i}$ they intend to send.
(b) Single Signature Verification: The verifier, holding the signature public key $Q_{i}$, receives the message $m_{i}$ and uses the public key $Q_{i}$, the message $m_{i}$, and the signature $\sigma_{i}$ to perform the verification.
(c) Aggregate Signature: The aggregator, holding the signature public keys $Q_{i}$ for multiple messages, the signatures $\sigma_{i}$ of these multiple messages, and other accompanying information $om_{i}$ sent, aggregates the signatures to obtain an aggregate signature σ along with the packaged messages M and accompanying information OM.
(d) Aggregate Signature Verification: The verifier, upon receiving the aggregate signature σ, the packaged messages M, and the accompanying information OM sent by the aggregator, uses the corresponding public keys $Q_{i}$ to perform the verification.

These steps are the most time-consuming parts of the aggregate signature mechanism. This is particularly true when an error occurs in the aggregate signature, as it requires individual verification of each signature [26], leading to a significant amount of time spent on single signature verifications. 

### 2.3. K-CAA Problem

At present, the main computational hardness assumption problems for elliptic curves include the Computational Diffie–Hellman (CDH) problem [25] and ECDLP [3]. In elliptic curve aggregate signatures, the commonly used hardness assumption is the K-CAA problem. Below is the definition of the K-CAA problem. 

Let $\left(G_{1},+\right)$ be a cyclic group of order q, where q is a large prime number. For an integer k and unknown $x\in Z_{q}^{*}$, given $\left\{e_{1},e_{2},\cdots ,e_{k}\right\}\in Z_{q}^{*}$, $g,xg\in G_{1}$ and $\left\{\frac{1}{x+e_{1}}g,\frac{1}{x+e_{2}}g,\cdots ,\frac{1}{x+e_{k}}g\right\}$, compute $\left(e,\frac{1}{x+e}g\right)$, where $e\in Z_{q}^{*},e\notin \left\{e_{1},e_{2},\cdots ,e_{k}\right\}$.

### 2.4. Fault Tolerance Mechanism

To address the significant time consumption caused by verifying individual signatures due to aggregated erroneous signatures, Hartung et al. [9] proposed the concept of fault-tolerant aggregate signatures and conducted theoretical derivations. References [27,28] further extended these schemes based on non-overlapping sets using compression and nesting methods, respectively. However, these schemes are very challenging to implementing in practice. Wang et al. [26] proposed a new fault-tolerant scheme based on uniform(k,n)−set combinatorial theory and validated it theoretically. We adopt uniform(k,n)−set approach. Below is an introduction to the theoretical framework. Given a set $A=\left\{a_{1},a_{2},\ldots ,a_{m-1},a_{m}\right\}$ and its subsets $A_{1},A_{2},\ldots A_{n-1},A_{n}\subset A$, these subsets satisfy uniform(k,n)−set, where $m\geq C_{n}^{k-1}$. Additionally, these subsets meet the following conditions: (1) The sizes of these subsets are all equal. (2) For any k subsets $A_{i1},A_{i2},\ldots A_{ik}\in \left\{A_{1},\ldots A_{n-1},A_{n}\right\}$, the union of $A_{i1},A_{i2},\ldots A_{ik}$ is A. (3) For any k−1 distinct subsets, the union of any $A_{ij},j\in \left[1,k-1\right]$ subsets contains at least one element $a_{i}$ that is not included in the union.

Cao et al. [29] proposed a method suitable for constructing a structure of uniform(k,n)−set. First, the set $A_{1},A_{2},\ldots A_{n-1}$ is evenly divided into $m=C_{n}^{k-1}$ groups, each including k−1 subsets $A_{i}$. The format of the subsets is shown in Equation (1).

(1) $$\left\{\begin{array}{c} W_{1}=A_{11}\cup A_{12}\ldots \cup A_{1k-1}\\ \ldots \\ W_{m}=A_{m1}\cup A_{m2}\ldots \cup A_{mk-1} \end{array}\right.$$

where $A_{i1}\in A,i=1,2,\ldots ,m,j=1,2,\ldots ,k-1,i\neq j,W_{i}\neq W_{j},a\neq b,a_{b}\notin W_{b},a_{b}\notin W_{c},1\leq b,c\leq m$.

Below is an example. Suppose we have a set $A=\left\{a_{1},\ldots ,a_{10}\right\}$; then, m=10. It is straightforward to set n=5, k=4, which satisfies the condition $C_{n}^{k-1}=10$. Then, dividing these 5 subsets $A_{1},\ldots ,A_{5}$ into 10 groups can be achieved by arranging them into 10 different sets of numbers, that is, $\left\{A_{1},A_{2},A_{3}\right\},\;\left\{A_{1},A_{2},A_{4}\right\},\;\left\{A_{1},A_{2},A_{5}\right\},\cdots ,\left\{A_{3},A_{4},A_{5}\right\}$.

Based on the requirements for uniform(k,n)−set mentioned above, we can derive the specific subsets $A_{1},\ldots ,A_{5}$, as shown in Equation (2):

(2) $$\begin{array}{l} A_{1}=\left\{a_{1},a_{2},a_{4},a_{7}\right\}\\ A_{2}=\left\{a_{2},a_{3},a_{6},a_{9}\right\}\\ A_{3}=\left\{a_{1},a_{3},a_{5},a_{8}\right\}\\ A_{4}=\left\{a_{4},a_{5},a_{6},a_{10}\right\}\\ A_{5}=\left\{a_{7},a_{8},a_{9},a_{10}\right\} \end{array}$$

The specific construction method is referred to in references [26,29].

## 3. Entire Structure of the Scheme

In our work, there are two available modes: the classic aggregate signature mode and the fault-tolerant aggregate signature mode. The classic aggregate signature mode is suitable for scenarios with minimal network errors, while the fault-tolerant aggregate signature mode can handle situations with a certain error rate. The choice between these two methods only requires the Road-Side Unit (RSU) to decide based on its own situation with little impact on the perception of the authentication center and vehicles in the network. 

**Figure 2:**
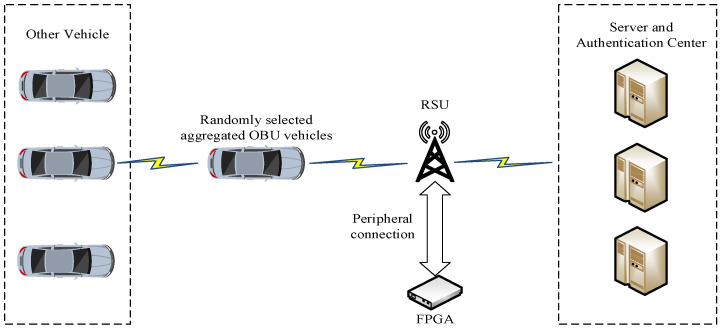
The overall structure of the efficient SM9 aggregate signature scheme.

As shown in Figure 2, the entire scheme is illustrated. While vehicles are driving on the road, they collect information, which is then aggregated by a randomly selected vehicle responsible for gathering the aggregated information, and subsequently sent to the RSU. The RSU initially processes the information using its CPU before passing it to the attached FPGA device for further processing. After the FPGA processes the information, it is sent back to the CPU for final processing. Once the RSU has processed the information, it is fed back to the vehicle cluster and the server. This scheme is an improvement based on the systems described in references [4,30]. The processes of system establishment, master key generation, vehicle registration, and key distribution are the same as those in the aforementioned references. The following sections will describe the main parts and the improved processes, including the sign, single signature verification, aggregate signature generation, and aggregate signature verification.

First, the main parameters required for the efficient SM9 aggregate signature scheme need to be explained, as shown in Table 1. The public key, private key, and pseudonym of the vehicle are assigned by the authentication center.

The following is the overall process of the efficient SM9 aggregate signature scheme. This section is divided into four parts: sign, single signature verification, fault-tolerant aggregate signature, and fault-tolerant aggregate verification.

(1) Sign: $sign(m_{i},VID_{i})$, where $m_{i}$ is the message to be sent.
  (a) Compute $g=e\left(P_{1},kpb\right)$;
  (b) Randomly select $r_{i},r_{i}\in Z_{n-1}^{*}$, and compute $w_{i}=g^{r_{i}}$;
  (c) Compute $h_{2i}=H_{2}\left(m_{i}||w_{i},N\right),L_{i}$. If $L_{i}=0$, reselect the random number; otherwise, $L_{i}=\left(r_{i}-VID_{i}\right)\mathrm{mod}N$;
  (d) Compute $S_{i}=L_{i}\cdot s_{i},U_{i}=L_{i}\cdot s_{i}\cdot VID_{i}$;
  (e) Package and send $\left(m_{i},S_{i},U_{i},w_{i}\right)$.
(2) Single signature verification: To verify the received message packet $\left(m_{i},S_{i},U_{i},w_{i}\right)$, the verification process is as follows:
  (a) Compute $g=e\left(P_{1},kpb\right),h_{2i}=H_{2}\left(m_{i}||w_{i},N\right),t_{i}=g^{h_{2i}}$;
  (b) Compute $v_{i}=e\left(S_{i},Q_{i}\right)\cdot t_{i}$, verify whether $v_{i}$ is equal to $w_{i}$. If they are not equal, the verification fails.
(3) Fault-tolerant aggregate signature: The RSU can negotiate with the selected vehicle OBU based on its own situation to adopt the following non-fault-tolerant or fault-tolerant aggregate signature modes.
  (a) Non-fault-tolerant aggregate signature mode;

The OBU of the randomly selected vehicle acts as the signature aggregator. The set of pseudonyms of the received vehicles is $\left\{VID_{1},\ldots ,VID_{n}\right\}$, the corresponding set of messages is $\left\{m_{1},\ldots m_{n}\right\}$, and the received digital signatures are $\left\{\left(S_{1},U_{1},w_{1}\right),\ldots ,\left(S_{n},U_{n},w_{n}\right)\right\}$. The signature aggregator computes $NU=\sum_{i=1}^{8}U_{i},NS=\sum_{i=1}^{8}S_{i},NW=\sum_{i=1}^{8}w_{i}$. The aggregate signature is $\delta =\left(NS,NU,NW,w_{1},\ldots ,w_{8}\right)$.

 
  (b) Fault-tolerant aggregate signature mode.

The OBU of the randomly selected vehicle acts as the signature aggregator, performing fault-tolerant aggregation processing on the received signature set. The set of pseudonyms of the received vehicles is $\left\{VID_{1},\ldots ,VID_{n}\right\}$, the corresponding set of messages is $\left\{m_{1},\ldots m_{n}\right\}$, and the received digital signatures are $\left\{\left(S_{1},U_{1},w_{1}\right),\ldots ,\left(S_{n},U_{n},w_{n}\right)\right\}$. Due to the limited resources of the RSU, the received digital signatures and messages are divided into uniform(8,9). According to the lemma mentioned in [26], there are a total of nine subsets, each containing eight elements. These are defined as $\left\{T_{1},\ldots ,T_{9}\right\}$. The fault-tolerant aggregate signature set composition is represented as $T_{i}=\left(TS_{i},TU_{i},Tw_{i},w_{1},\ldots ,w_{8}\right)$, where $w_{1},\ldots ,w_{8}$ is not specifically the first eight received digital signatures but rather a generic representation. Suppose the digital signature set $\left\{\left(S_{1},U_{1},w_{1}\right),\ldots ,\left(S_{8},U_{8},w_{8}\right)\right\}$ forms the combination elements of uniform(8,9); then, the aggregation method of $T_{i}$ is shown in Equations (3)–(5).

(3) $$TS_{i}=\sum_{i=1}^{8}S_{i}$$

(4) $$TU_{i}=\sum_{i=1}^{8}U_{i}$$

(5) $$Tw_{i}=\prod_{i=1}^{8}w_{i}$$

(4) Fault-tolerant aggregate verification: The RSU selects the verification mode based on the previously negotiated mode.
  (a) Non-fault-tolerant verification mode;

The RSU receives the message set $\left\{m_{1},\ldots m_{n}\right\}$ and the aggregate signature $\delta =\left(NS,NU,NW,w_{1},\ldots ,w_{8}\right)$ sent by the vehicle OBU, computation $h_{2i}=H_{2}\left(m_{i}||w_{i},N\right)$ and verification Equation (6).

(6) $$e\left(NS,kpb\right)e(NU,P_{2})e(\sum_{i=1}^{8}h_{2i}P_{1},kpb)=NW$$

 
  (b) Fault-tolerant verification mode.

After receiving the message set $\left\{m_{1},\ldots m_{n}\right\}$ and the fault-tolerant aggregate signature $\left\{T_{1},\ldots ,T_{n}\right\}$ sent by the signature aggregator, the RSU computes $h_{2i}=H_{2}\left(m_{i}||w_{i},N\right)$ and verifies each fault-tolerant aggregate signature using Equation (7), where c is the number of signatures that constitute a single fault-tolerant aggregate signature set.

(7) $$e\left(TS_{i},kpb\right)e(TU_{i},P_{2})e(\sum_{i=1}^{c}h_{2i}P_{1},kpb)=Tw_{i}$$

It can be noted that there are a large number of point multiplication operations in Equations (6) and (7). Regardless of whether it is in the non-fault-tolerant aggregation or fault-tolerant aggregation process, the number of point multiplication operations will increase with the number of signatures, and point multiplication is a quite time-consuming operation [31]. Therefore, the time consumption will surge. Consequently, the point multiplication operations in the SM9 efficient aggregate signature scheme can be offloaded to the FPGA board for computation.

## 4. Hardware Improved Acceleration Structure

**Figure 3:**
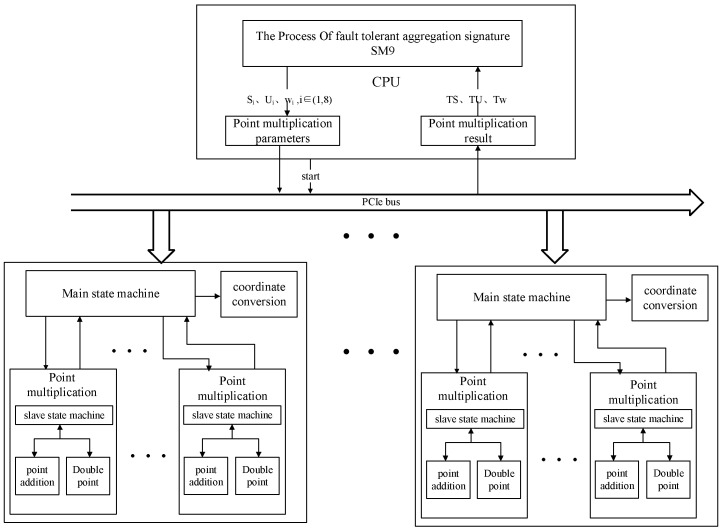
Hardware acceleration overall structure.

The overall structure of the hardware acceleration for the efficient SM9 aggregate signature scheme is shown in Figure 3. The architecture consists of a CPU connected to multiple FPGA boards with information exchanged via the PCIe bus. The CPU is responsible for bilinear pairing operations involved in the SM9 algorithm, the configuration of parallel point multiplication parameters in SM9, and the final verification of the results. The FPGA boards are responsible for parallel acceleration of the point multiplication operations.

**Figure 4:**
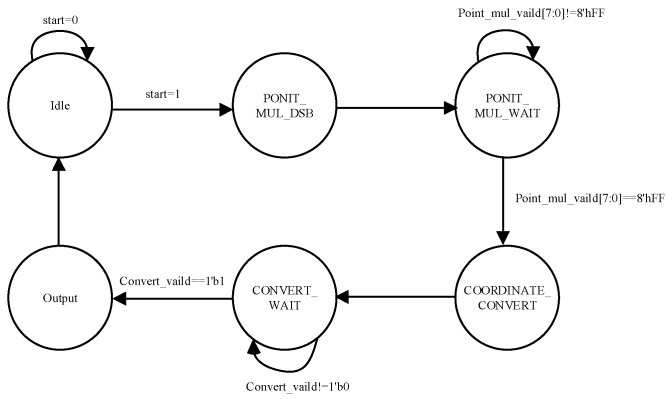
The figure of master state machine state transition.

As illustrated in Figure 3, a master state machine is set up to control the data flow within each point multiplication unit and handle the subsequent processing of the computation results. The state transition diagram of the master state machine is shown in Figure 4.

In the Idle state, parameters are read, and the state machine is initialized. When the start signal is valid, the state machine updates multiple point multiplication parameter registers and transitions to the PONIT_MUL_DSB state; otherwise, it remains in Idle. Upon transitioning to the PONIT_MUL_DSB state, the state machine reads the multiple point multiplication tasks and assigns each individual point multiplication task to the corresponding point multiplication unit. Once the task distribution is completed, the state transitions to the PONIT_MUL_WAIT state. In the PONIT_MUL_WAIT state, after all point multiplication tasks are completed, the state machine reads all the computed results and transitions to the COORDINATE_CONVERT state. In the COORDINATE_CONVERT state, the state machine transfers all point multiplication results to the coordinate conversion unit for merging and coordinate transformation. Then, it transitions to the CONVERT_WAIT state. While in the CONVERT_WAIT state, the state machine waits for the coordinate conversion unit to complete its computation. After the coordinate conversion is completed, the state machine reads the results and transitions to the Output state to output the computed results. Finally, the state transitions back to the Idle state.

For each point multiplication unit, the state machine is responsible for controlling the data flow between the point addition and point doubling units, achieving unit reuse. Additionally, by configuring multiple point multiplication modules, the on-board resources can be fully utilized to accelerate large-scale point multiplication operations.

### 4.1. Montgomery Point Multiplication

Montgomery point multiplication is currently the most efficient point multiplication algorithm, with a time complexity superior to existing methods such as binary expansion, non-adjacent form (NAF), and NAF-related improved algorithms. Montgomery point multiplication requires a base point G, and subsequently calculates $P_{1}$ and $P_{2}$, where $P_{1}=G,P_{2}=2G$. Then, based on $P_{1}$, $P_{2}$ and the multiplication scalar k, the point multiplication result is computed. The computation process is shown in Algorithm 1. Additionally, it can resist simple power analysis attacks.
**Algorithm 1: Montgomery Point Multiplication**Input:
$G=(x_{G},y_{G}),k=\left(k_{n-1},\ldots ,k_{0}\right)$
Output:
Q=kG
1.$P_{1}=G;P_{2}=2G;i=n-2$2.while(i≥0) do3.$\;\;\mathrm{if}(k_{i}==0)$ then $P_{2}=P_{1}+P_{2};P_{1}=2P_{1}$;4.$\;\;\mathrm{else}\;\mathrm{if}\;(k_{i}==1)$ then $P_{1}=P_{1}+P_{2};P_{2}=2P_{2}$;5.  i=i−1;6.  end if7.end while8.$Q=P_{1}$

### 4.2. Optimization of Finite Field Operational Units

Finite field arithmetic units are the fundamental components of elliptic curve operations, and they include modular addition, modular subtraction, modular multiplication, and modular inversion modules. The performance of these finite field arithmetic units is crucial for the computation speed of point multiplication algorithms on the FPGA.

#### 4.2.1. Design of Modular Addition and Subtraction Units

To optimize the use of on-board resources and reduce the area, modular addition and subtraction are implemented in a combined manner using pure combinational logic. This module performs calculations based on the initial input modulus P and mode selection mode. Since modular addition can result in overflow and modular subtraction can result in negative outcomes, these situations need to be handled accordingly. The optimized computation method is shown in Algorithm 2.
**Algorithm 2: Modular Addition and Subtraction Algorithm**Input:
A,B,P,mode
Output:
C
1.  if(mode==0)begin2.  
$B_{1}=B,Bam=P$
3.  end else begin4.  
$B_{1}=P,Bam=B$
5.  end6.  $\left\{CA_{1},CA_{2}\right\}=A+B_{1}$
7.  $\left\{CAS_{1},CAS_{2}\right\}=\left\{CA_{1},CA_{2}\right\}-\left\{1\prime b0,Bam\right\}$
8.  $\left\{CS_{1},CS_{2}\right\}=A-B$
9.  if(mode==0)begin10.  $\mathrm{if}(CAS_{1}==1)$begin11.  
$C=CA_{2}$
12.  end else begin13.  
$C=CAS_{2}$
14. end 15. end else begin16.  $\mathrm{if}(C_{2}==1)$begin
17.   $C=CAS_{2}$
18.  end else begin19.   
$C=CS_{2}$
20.  end 21. end

**Figure 5:**
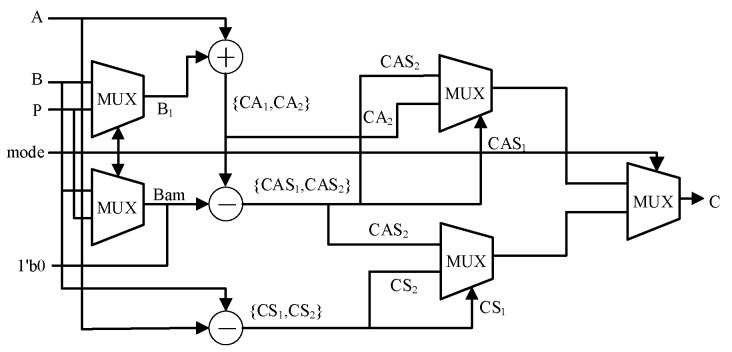
The figure of modular addition and subtraction units.

As shown in Figure 5, the hardware structure of the modular addition and subtraction is composed of pure combinational logic, forming a parallel circuit structure. The computation is completed within a single clock cycle.

#### 4.2.2. Design of the Modular Inversion Unit

Currently, the algorithms for computing modular inverses include Fermat’s Little Theorem, the Montgomery algorithm, the Extended Euclidean algorithm, and the binary modular inversion algorithm. Fermat’s Little Theorem requires modular exponentiation, while the Montgomery algorithm needs two domain transformations to obtain the final modular inverse result. Additionally, the original Extended Euclidean algorithm involves division in each step of the multiplication operations, which is highly costly [32]. Therefore, a more hardware-suitable binary modular inversion algorithm is adopted. The computation process of the binary modular inversion algorithm is shown in Algorithm 3.
**Algorithm 3: Modular Inverse Algorithm**Input:
a,b,p
Output:
$c=ba^{-1}\mathrm{mod}p$
1.  
u=a,v=p,x1=b,x2=0
2.  while(u!=1&&v!=1) do3.  if(u[0]==0&&x1[0]==0) then4.   u=u>>>1; $x_{1}=x_{1}>>>1$;5.   end if6.  if(u[0]==0&&x1[0]==1) then7.   u=u>>>1; $;x_{1}=\left(x_{1}+p\right)>>>1$;8.  end if9.  if(u[0]==1&&v[0]==0&&x2[0]==0) then10.   v=v>>>1; $x_{2}=x_{2}>>>1$;11. end if12.  if(u[0]==1&&v[0]==0&&x2[0]==1) then13.   v=v>>>1; $x_{2}=\left(x_{2}+p\right)>>>1$;14.  end if15.  if(u[0]==1&&v[0]==1) then16.      if (u≥v) then u=u−v; $x_{1}=x_{1}-x_{2}$;else17.         v=v−u; $x_{2}=x_{2}-x_{1}$;18.      end if19  end if20.  end while21.  if (u!=1) then $x_{1}==x_{2}$ end if22.  $\mathrm{while}(x_{1}\geq p$) do23.    
$x_{1}=x_{1}-p$
24.  end while25.  $c=x_{1}$;

#### 4.2.3. Design of the Fast Modular Multiplication Unit

In the computation of elliptic curve finite fields, modular multiplication is the most critical part affecting performance. Modular multiplication includes the Montgomery multiplication algorithm, interleaved modular multiplication algorithm, and Barrett modular multiplication algorithm. Although Montgomery multiplication offers good generality and flexibility, it requires a domain transformation of data. The interleaved modular multiplication involves many iterations, leading to longer computation time. The Barrett modular multiplication algorithm, on the other hand, can achieve low-cost computation for any modulus p with precomputed parameters. Therefore, this section uses and Barrett modular reduction algorithm to implement the Barrett fast modular reduction algorithm.

(1) KOA Fast Multiplication

The main idea of KOA multiplication is to use a recursive approach to reduce multiple lower-complexity multiplications into a higher-complexity multiplication. This results in a faster and more efficient multiplication algorithm. In the computation of KOA, some parameters need to be defined first. For an n bit number A, it can be represented in binary as $A=\left(a_{n-1},\cdots ,a_{1},a_{0}\right)$, $a_{i}\in \left\{0,1\right\},0\leq i\leq n-1$, where $A_{H}=\left(a_{n-1},\cdots ,a_{n/2}\right)$ is the higher-order part and $A_{L}=\left(a_{n/2-1},\cdots ,a_{0}\right)$ is the lower-order part. Using the above definitions, the two numbers A and B to be multiplied using KOA multiplication can be represented as

(8) $$A=A_{H}\times 2^{n/2}+A_{L}$$

(9) $$B=B_{H}\times 2^{n/2}+B_{L}$$

For C=A×B, the result of the computation is

(10) $$C=A_{H}\times B_{H}\times 2^{n}+\left(A_{H}\times B_{L}+A_{L}\times B_{H}\right)\times 2^{n/2}+A_{L}\times B_{L}$$

while $A_{H}\times B_{L}+A_{L}\times B_{H}$ can be represented as

(11) $$A_{H}\times B_{L}+A_{L}\times B_{H}=\left(A_{H}+A_{L}\right)\times \left(B_{H}+B_{L}\right)-A_{H}\times B_{H}-A_{L}\times B_{L}$$

From the above, it is clear that KOA multiplication only requires the computation of $A_{H}\times B_{H}$, $A_{L}\times B_{L}$ and $\left(A_{H}+A_{L}\right)\times \left(B_{H}+B_{L}\right)$, further reducing the computational complexity.

**Figure 6:**
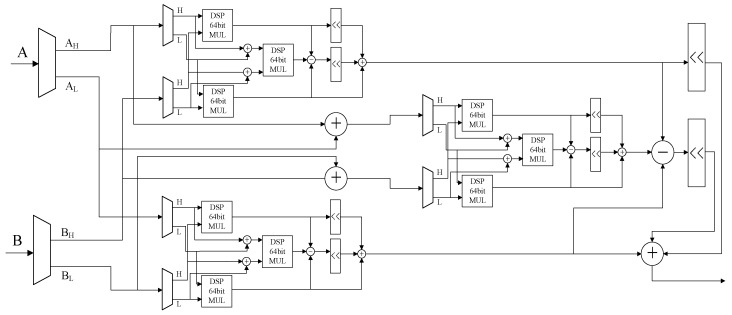
Overall architecture of KOA 256 bit algorithm.

In SM9, all operations are based on 256-bit numbers. To better utilize the FPGA’s on-board resources, a double recursion method can be used to divide a 256-bit multiplication into multiple 64-bit multiplications. Additionally, the multiplications $A_{H}\times B_{H}\times 2^{n}+\left(A_{H}\times B_{L}+A_{L}\times B_{H}\right)\times 2^{n/2}$ of $2^{n}$ and $2^{n/2}$ can be accomplished through bit shifting. The KOA multiplication process is shown in Algorithm 4.
**Algorithm 4: KOA Multiplication**Input: A,B,n,Output:
C
1.  if(n==64) then return
C=A×B
2.  end if3.  $A=A_{H}\times 2^{n/2}+A_{L}$;4.  $B=B_{H}\times 2^{n/2}+B_{L}$;5.  $X=KOA\left(A_{H},B_{H},\frac{n}{2}\right)$;6.  $Y=KOA\left(A_{H}+A_{L},B_{H}+B_{L},\frac{n}{2}\right)$;7.  $Z=KOA\left(A_{L},B_{L},\frac{n}{2}\right)$;8.  D=Y−X−Z;9.  $C=X<<n+D<<\frac{n}{2}+Z$;

To further leverage the performance of the KOA algorithm on the FPGA, the computation cycle of the DSP 64-bit multipliers used in the component design is set to 0. Wire-type variables are used to connect the components within the module, achieving the goal of immediate output upon input. A register is then set at the end to divide the clock cycle. As shown in Figure 6, the schematic diagram of the 256-bit KOA algorithm expansion is illustrated, where H and L represent the higher-order and lower-order bits of the further split data. Finally, a 512-bit multiplication result is output through an adder.

(2) Barret Fast Multiplication

For any two positive integers A and B, there exist two numbers q and r such that the following equation holds:

(12) $$A=q\times B+r,r\in \left[0,b-1\right]$$

Thus, A=rmodB, where B is the modulus for the modular operation. However, finding such numbers q and r requires high-cost division operations. But when the modulus is a fixed value, the Barrett algorithm can be used to perform modular reduction using multiplication and shift operations [33]. Let $a,b\in \left[0,P-1\right]$ to obtain the result of a×bmodP. We can set d=a×b, then d=e×P+c, and we have c, which is the result of a×bmodP.

First, we calculate the approximate value $e_{1}$ of e. Let $\mu =\left\lfloor \frac{2^{2n}}{P}\right\rfloor ,e_{1}=\left\lfloor \frac{d}{P}\right\rfloor$, where n is the bit length of the modulus P in its binary representation. Then, $e_{1}=\left\lfloor \frac{d}{P}\right\rfloor \approx \left\lfloor \left\lfloor \frac{d}{2^{n}}\right\rfloor \frac{\mu }{2^{n}}\right\rfloor =\left\lfloor \left\lfloor d>>n\right\rfloor \mu >>n\right\rfloor$. The computation of $e_{1}$ thus transforms into a combination of shifts and multiplications. For the calculation of μ, this value is precomputed in software and then input μ into the FPGA. In practical applications, once the modulus P is selected, it is typically treated as a system constant. Therefore, μ can also be treated as a system constant. The Barrett modular reduction process is shown in Algorithm 5.
**Algorithm 5: Barrett Reduction**Input: a,b,n,P,μ,Output:
c=a×bmodP
1.  d=a×b;2.  $e_{11}=\mu \left[n-1:0\right]\times d/2^{n}$;3.  $\mathrm{if}(\mu \left[n\right]==1\prime b1$)then4.  $e_{12}=\left\{\left(d>>n\right),256b\prime 0\right\}$;5.  $\mathrm{else}e_{12}=512\prime b0$;6.  end if7.  $e_{1}=\left(e_{11}+e_{12}\right)>>n$; 8.  $d_{1}=P\times e_{1}$; 9.  $c=d-d_{1}$; 10. if(c>P) then c=c−P; 11. end if 12. return c;

For the multiplications involved, KOA multiplication is used. Since μ can be up to 257 bits, steps 3 to 6 handle this case. The final computed c, as it ranges from 0 to 2P−1, needs to be checked and adjusted if necessary. Steps 9 to 11 handle this process.

### 4.3. Optimization of Point Addition and Point Double Modules

In elliptic curve point multiplication, the use of different coordinate systems results in varying computational complexities. For instance, operations in the affine coordinate system involve modular inversion, which is computationally expensive. In our work, we employ the standard projective coordinate system for the computations. In the standard projective coordinate system, modular inversion is required only when converting back to the affine coordinate system. Additionally, in the Montgomery point multiplication process within the standard projective coordinate system, computations are simplified as they only need to be performed on the coordinates X and Z, thus reducing the computational burden.

Consider points $P_{1}=\left(X_{1},Y_{1},Z_{1}\right),\;P_{2}=\left(X_{2},Y_{2},Z_{2}\right)$ and $P_{3}=\left(X_{3},Y_{3},Z_{3}\right)$ in the standard projective coordinate system. The point addition formulas are given by Equations (13) and (14).

(13) $$X_{3}={\left(X_{1}X_{2}-aZ_{1}Z_{2}\right)}^{2}-4bZ_{1}Z_{2}\left(X_{1}Z_{2}+X_{2}Z_{1}\right)$$

(14) $$Z_{3}=x_{G}{\left(X_{1}Z_{2}-X_{2}Z_{1}\right)}^{2}$$

The point double operation formulas are given by Equations (15) and (16).

(15) $$X_{3}={\left(X_{1}^{2}-aZ_{1}^{2}\right)}^{2}-8bX_{1}Z_{1}^{3}$$

(16) $$Z_{3}=4Z_{1}\left(X_{1}^{3}+aX_{1}Z_{1}^{2}+bZ_{1}^{3}\right)$$

After completing the computations, the results are converted back to the affine coordinate system. The conversion methods are given by Equations (17) and (18).

(17) $$x_{i}=\frac{X_{i}}{Z_{i}},i\in \left[1,3\right]$$

(18) $$y_{3}=\frac{2b+\left(a+x_{3}x_{G}\right)\left(x_{3}+x_{G}\right)-x_{1}{\left(x_{3}-x_{G}\right)}^{2}}{2y_{G}}$$

The resulting $\left(x_{3},y_{3}\right)$ are the converted results in the affine coordinate system.

#### Data Flow Optimization

To maximize the efficient use of on-board resources and complete point addition and point double in the shortest possible time, we optimize the data flow for state machine operations and Montgomery point multiplication. The computation processes and data flows for point addition and point double are shown in Table 2 and Table 3, respectively.

In the design presented in our work, the point addition and point double modules operate in parallel during the point multiplication process. Therefore, the total computation cycle count is determined by the module with the longest cycle duration. Modules with shorter cycles will idle, ensuring synchronization and smooth operation of the point multiplication state machine. Additionally, the multiplication operations utilize a fast modular multiplication unit with a computation cycle of seven cycles, while the modular addition and subtraction units each have a computation cycle of one cycle. Due to the presence of the state machine and synchronization stages, both the point addition and point double modules have an operation cycle of 102 cycles.

### 4.4. Optimization of Coordinate Transformation

After the point multiplication operation, the results need to be aggregated and coordinate conversion must be performed. In the design of this module, a modular inversion module and a point addition module are used with a state machine controlling the data flow and the reuse of these modules. The coordinate conversion method is described in Algorithm 6, where $X_{i},Z_{i},i\in \left[0,7\right]$ are the results output by the eight parallel point multiplication modules. The function Point_add is a wrapper for the point addition module, and $inv\left(a,b,p\right)$ is a wrapper for the modular inversion module. The output result is a/bmodP.
**Algorithm 6: Coordinate Transformation**Input: $\left(X_{i},Z_{i}\right),i\in \left[0,7\right],a,b,n,P,x_{G}$,Output:
x,y
1.  i = 1;
2.  $X,Z=X_{1},Z_{1}$
3.  while(i≤6) do4.  $X,Z=\mathrm{Point}\_\mathrm{add}(X,Z,X_{i+1},Z_{i+1},a,b,P,x_{G}$)5.  i = i+1;6.  end while7.
$x_{6}=inv\left(X_{6},Z_{6},P\right)$
8.
$x=inv\left(X,Z,P\right)$
9.
$T_{1}=x_{G}x$
10.
$T_{2}=x_{G}+x$
11. $T_{3}=x_{G}-x$;12. $T_{3}={\left(T_{3}\right)}^{2}$;13.
$T_{3}=x_{6}T_{3}$
14.
$T_{1}=T_{1}+a$
15.
$T_{1}=T_{1}T_{2}$
16.
$T_{1}=T_{1}+b$
17.
$T_{1}=T_{1}+b$
18.
$T_{1}=T_{1}-T_{3}$
19.
$T_{1}=T_{1}T_{2}$
20.
$y=y_{G}+y_{G}$
21.
$y=inv\left(y,T_{1},P\right)$
22.
$\mathrm{return}\left(x,y\right)$


## 5. Experimental Results and Scheme Analysis

The host machine in this study is a computer with a Pentium Dual CPU E2200 2.20 GHz running Ubuntu 16.04, which can support simultaneous connections to nine FPGA boards. The hardware platform consists of a system of nine FPGA boards, where hardware modules are designed using the Verilog language based on FPGA boards with the chip model xcku-060-ffva1156-2-i. The software used is Vivado 2019.2.

### 5.1. Safety Analysis

#### 5.1.1. Security Proof

For a signature scheme, its security must satisfy existential unforgeability under adaptive chosen message attacks (EUF-CMA). To prove that the proposed scheme possesses this property, the following definitions and proof process are provided. To address the security of the scheme, consider an attacker who does not have the capability to learn the master private key but can replace the vehicle’s public key. Under this attacker’s threat model, to prove that the scheme satisfies existential unforgeability under adaptive chosen message attacks (EUF-CMAs), Game 1 is defined as follows.

**Definition** **1:**  *Facing a Type 1 attacker, if no Type 1 attacker can win Game 1 with a non-negligible advantage, then the proposed scheme is EUF-CMA secure.*

**Game** **1:**  *Let C be the challenger, $A_{1}$ be the Type 1 attacker, and $ID_{i}$ be the target being challenged. The game is constructed as follows:*

(1) System Initialization: The system initialization is executed by C, generating the system parameter set Parms and the master private key ks. The system parameter set Parms is then sent to the attacker $A_{1}$, while the master private key ks is kept secret.
(2) First Phase Queries: In this phase, $A_{1}$ can make the following queries to C: hash queries, private key queries, and signature queries.
  (a) Private Key Query: When $A_{1}$ requests the private key for $ID_{i}$, C sends the private key to $A_{1}$.
  (b) Signature Query: When $A_{1}$ requests this query, assuming the requested parameters are $\left(ID_{i},m_{i}\right)$, C sends the signature to $A_{1}$. However, $A_{1}$ cannot request a signature query where $ID_{i}$ is the identity of the forger; otherwise, the game terminates.
(3) Forgery: The attacker $A_{1}$ forges a digital signature $\left(S_{i},U_{i},w_{i}\right)$ for the identity $ID_{i}$. $A_{1}$ is considered to have won Game 1 if the following conditions are met:
  (a) The signature $\left(S_{i},U_{i},w_{i}\right)$ is valid.
  (b) The master private key has not been queried.
  (c) In the signature query, the requested parameters do not include the identity $ID_{i}$.

**Theorem** **1:**  *In the random oracle model, if the K-CAA problem is hard to solve, then the proposed scheme is EUF-CMA secure.*

**Lemma** **1:**  *In the random oracle model, if there exists a Type 1 attacker $A_{1}$ who can win Game 1 with non-negligible advantage ε (after making at most $q_{H_{i}}\left(i=1,2\right)$ $H_{i}$ hash queries, $q_{E}$ private key queries, and $q_{s}$ signature queries), then there exists a challenger C who can solve the K-CAA problem with non-negligible advantage $succ_{A_{1}}^{K-CAA}\geq \left(\varepsilon -\frac{1}{2^{k}}\right){\left(\frac{q_{E}}{q_{H_{1}}}\right)}^{q_{E}+q_{S}}\left(\frac{q_{H_{1}}-q_{E}}{q_{H_{1}}}\right)$*.

**Proof:**  Game 1 is constructed between the challenger C and the attacker $A_{1}$. If $A_{1}$ can forge a valid signature, then C can leverage Game 1 to solve the K-CAA problem. Therefore, given random inputs $R=kP_{2}\in G_{1},h_{1}=H_{1}\left(ID_{i}||hid,N\right),h_{1_{i}},\ldots ,h_{1_{q_{E}}}\in Z_{q}^{*}$ and $\frac{h_{1_{1}}P_{1}}{k+h_{1_{1}}},$ $\frac{h_{1_{2}}P_{1}}{k+h_{1_{2}}},\ldots ,\frac{h_{1_{q_{E}}}P_{1}}{k+h_{1_{q_{E}}}}$, the goal is to output a solution to the K-CAA problem where the solution is $\left(h_{1_{I}},\frac{h_{1_{I}}P_{1}}{k+h_{1_{I}}}\right),h_{1_{I}}\notin \left\{h_{1_{1}},h_{1_{2}},\ldots ,h_{1_{qE}}\right\}$.

(1) System Initialization: The system initialization is executed by C. Let $kpb=R=kP_{2}$, where ks=k serves as the master private key, which is unknown to C. The challenger C selects an identity $ID_{i}$ as the challenge identity and sends the parameters $\left\{P_{1},P_{2},kpb,H_{1},H_{2}\right\}$ to the attacker $A_{1}$.
(2) First Phase Queries: $A_{1}$ can initiate queries to C, and C must respond according to the game rules. The possible queries include $H_{1}$ hash queries, $H_{2}$ hash queries, random oracle queries, public key queries, private key queries, public key replacement queries, and signature queries. Additionally, lists $H_{1}^{List},H_{2}^{List},sk^{list}$ and $pk^{list}$ are used to store the records of $H_{1}$ hash queries, $H_{2}$ hash queries, random oracle queries, public key queries, and private key queries, respectively. The following details each type of query in the first phase:
  (a) $H_{1}$ Hash Query: When $A_{1}$ requests an $H_{1}$ hash query for input $\left(ID_{i},hid,N\right)$, C first searches the list $H_{1}^{List}$ to see if there is a corresponding tuple. The structure of the tuple $H_{1}^{List}$ is $\left(ID_{i},hid,N,h_{1_{i}}\right)$. If $H_{1}^{List}$ already contains the tuple $\left(ID_{i},hid,N,h_{1_{i}}\right)$, C directly returns $h_{1_{i}}$ to $A_{1}$. Otherwise, C randomly selects a value $h_{1_{i}}\in Z_{q}^{*}$, sets $h_{1_{i}}=H_{1}\left(ID_{i}||hid,N\right)$, forms a new tuple $\left(ID_{i},hid,N,h_{1_{i}}\right)$, inserts this tuple into the list $H_{1}^{List}$, and then returns $h_{1_{i}}$ to $A_{1}$.
  (b) $H_{2}$ Hash Query: When $A_{1}$ requests a $H_{2}$ hash query for input $\left(ID_{i},m_{i},r_{i},N\right)$, C first searches the list $H_{2}^{List}$ to see if there is a corresponding tuple. The structure of the tuple $H_{2}^{List}$ is $\left(ID_{i},m_{i},r_{i},w_{i},N,h_{2_{i}}\right)$. If $H_{2}^{List}$ already contains the tuple $\left(ID_{i},m_{i},r_{i},N,h_{2_{i}}\right)$, C directly returns $h_{2_{i}}$ to $A_{1}$. Otherwise, C randomly selects two values $r_{i},h_{2_{i}}\in Z_{q}^{*}$, sets $w_{i}=e{\left(P_{1},kpb\right)}^{r_{i}},h_{2_{i}}=H_{2}\left(m_{i}∥w_{i},N\right)$, forms a new tuple $\left(ID_{i},m_{i},r_{i},w_{i},N,h_{2_{i}}\right)$, inserts this tuple into the list $H_{2}^{List}$, and then returns $h_{2_{i}}$ to $A_{1}$. This $H_{2}$ hash query event is denoted by $E_{1}$.
  (c) Private Key Query: When $A_{1}$ requests the private key for identity $ID_{i}\left(1\leq i\leq q_{E}\right)$, C retrieves $\left(ID_{i},h_{1_{i}}\right)$ from the list $H_{2}^{List}$. C then checks if $h_{1_{i}}$ belongs to the challenge identity set $\left\{h_{1_{1}},h_{1_{2}},\cdots ,h_{1_{q_{E}}}\right\}$. If it is not, the game terminates (this event is denoted by $E_{2}$); otherwise, C sets $s_{i}=P_{1}-\frac{h_{1_{i}}P_{1}}{k+h_{1_{i}}},Q_{i}=\frac{kpbP_{1}}{s_{i}}$ and saves the tuple $\left(ID_{i},hid,N,Q_{i},s_{i}\right)$ in $sk^{list}$. C then returns $s_{i}$ and $Q_{i}$ to $A_{1}$.
(3) Forgery Phase: $A_{1}$ outputs a digital signature $\left(S_{I},U_{I},w_{I}\right)$ for the identity $ID_{I}$, and the signature passes the verification $e\left(S_{I},Q_{I}\right)\cdot e{\left(P_{1},kpb\right)}^{h_{2_{I}}}=e{\left(P_{1},kpb\right)}^{r_{I}}$.

C retrieves $\left(ID_{I},h_{1_{I}}\right)$ from $H_{1}^{list}$. Then, it retrieves the tuple $\left(ID_{i},m_{i},r_{i},w_{i},N,h_{2_{i}}\right)$ from the list $H_{2}^{list}$. Let $Q_{I}=h_{1_{I}}P_{2}+kpb$. If $h_{1_{I}}\in \left\{h_{1_{1}},h_{1_{2}},\cdots ,h_{1_{q_{E}}}\right\}$; then, the simulation terminates and outputs “Failure” (denoted as $E_{3}$), Otherwise, the equation $e\left(S_{I},Q_{I}\right)\cdot e{\left(P_{1},kpb\right)}^{h_{2_{I}}}=e{\left(P_{1},kpb\right)}^{r_{I}}$ holds, and let $x=\frac{h_{1_{I}}P_{1}}{k+h_{1_{I}}},Q_{I}=\frac{kpbP_{1}}{P_{1}-x}=kpbP_{1}{\left(P_{1}-\frac{h_{1_{I}}P_{1}}{k+h_{1_{I}}}\right)}^{-1}=kpbP_{1},{\left(\frac{kP_{1}}{k+h_{1_{I}}}\right)}^{-1}=h_{1_{I}}P_{2}+kpb$, so $e\left(S_{I},Q_{I}\right)$$=e\left(S_{I},\frac{kpbP_{1}}{P_{1}-x}\right)=e{\left(P_{1},kpb\right)}^{r_{I}-h_{2_{I}}},e\left(\frac{S_{I}P_{1}}{P_{1}-x},kpb\right)=e\left(\left(r_{I}-h_{2_{I}}\right)P_{1},kpb\right),\frac{S_{I}P_{1}}{P_{1}-x}=\left(r_{I}-h_{2_{I}}\right)P_{1}$. From this, we obtain $x=P_{1}-\frac{S_{I}}{r_{I}-h_{2_{I}}}=\frac{h_{1_{I}}P_{1}}{k+h_{1_{I}}}$, where $\left(h_{1_{I}},\frac{h_{1_{I}}P_{1}}{k+h_{1_{I}}}\right)$ is the solution to the K-CAA problem. Thus, C outputs the solution $\left(h_{1_{I}},\frac{h_{1_{I}}P_{1}}{k+h_{1_{I}}}\right)$ to the K-CAA problem. If the events $E_{1},E_{2}$ and $E_{3}$ do not occur, then C can solve an instance of the K-CAA problem. The probability that $A_{1}$ forges a valid signature without querying $H_{2}$ does not exceed $\frac{1}{2^{k}}$, so C’s probability of successfully solving the K-CAA problem is $succ_{A_{1}}^{K-CAA}\geq \left(\varepsilon -\frac{1}{2^{k}}\right){\left(\frac{q_{E}}{q_{H_{1}}}\right)}^{q_{E}+q_{S}}\left(\frac{q_{H_{1}}-q_{E}}{q_{H_{1}}}\right)$.

Because $A_{1}$’s advantage in winning the game is negligible, the probability of C successfully solving the K-CAA problem is negligible. Thus, the prerequisite for successfully solving the K-CAA problem does not exist. Furthermore, the fault-tolerant aggregate signature scheme constructed in our work operates mainly during the aggregation process and does not affect the aggregation method of the aggregate signature. Therefore, according to Definition 1, both the non-fault-tolerant and fault-tolerant aggregate signature schemes in our work are secure.

#### 5.1.2. Unforgeability

Based on the security of the signature scheme under the K-CAA assumption, the scheme proposed in this section can resist existential forgery under adaptive chosen message attacks and identity attacks. Therefore, the scheme presented in our work possesses unforgeability.

#### 5.1.3. Privacy

When a vehicle joins the IoV, it first sends its identity $ID_{i}$ to the authentication center to generate its pseudonym for use within the network. Consequently, during information exchange, no third party other than the authentication center can know the vehicle’s true identity. In subsequent communications, vehicles use pseudonyms to participate in the fault-tolerant aggregate signature process. Therefore, this scheme ensures the privacy of the vehicles participating in the communication.

#### 5.1.4. Traceability

When the RSU detects an error or verification failure in the aggregated signature, it can send the pseudonym of the vehicle that caused the verification error to the authentication center. The authentication center can then use the pseudonym to trace the relevant information of the vehicle. Therefore, this scheme has traceability.

### 5.2. Performance of Each Module

In the SM9 signature algorithm, the main time-consuming modules are bilinear pairing, modular exponentiation, and point multiplication. Among these, the most time-consuming step in the aggregate signature process is point multiplication. Bilinear pairing is implemented in software. The software computation times are as follows: bilinear pairing takes 4.129 ms, point multiplication takes 1.814 ms, and point addition takes 0.009 ms. These times are the average values obtained from running each operation 1000 times in software.

An eight-way parallel point multiplication algorithm is implemented on the FPGA. The specific details regarding the resource utilization, control method, operation mode, and operating cycles of each module are shown in Table 4. The test data are sourced from the SM9 Chinese National Standard [34].

Based on Table 4, from the perspective of structural functionality, the modular multiplication and modular inversion units in the parallel point multiplication algorithm are both computed serially. The modular addition and subtraction functions are combined using combinational logic, which completes the operations within a single clock cycle, exhibiting parallel characteristics. The point addition and point doubling units achieve their functionality by invoking the modular addition/subtraction and modular multiplication units. As seen from the table, point addition and point doubling are executed in parallel with their parallel nature supported by the Montgomery point multiplication algorithm and controlled by the slave state machine. Additionally, the table shows that the slave state machine and point multiplication modules also operate in parallel with their parallelism supported by the master control state machine, which simultaneously distributes tasks and manages parallel control. However, during coordinate conversion, all data from the point multiplication modules need to be sent to the master state machine, which then forwards it to the coordinate conversion module. The coordinate conversion module operates serially, and its serial nature is determined by the coordinate conversion algorithm.

According to Table 4, in terms of resource and performance analysis, a single-point multiplication module efficiently utilizes the FPGA’s board resources. When the eight-way parallel point multiplication algorithm is implemented, the overall resource utilization of each FPGA is LUT: 57.48%, FF: 23.38%, DSP: 93.91%. The operating frequency of the above modules is shown in Table 4 as well. The eight-way parallel point multiplication algorithm on the FPGA experimental platform achieves a calculation throughput of approximately $\left(2.7\times 10^{7}\right)/\left(26,014+1453\right)\times 8\times 9\approx 70,776$ operations per second. Meanwhile, the software implementation achieves only 551 operations per second, indicating a significant improvement in computational efficiency.

### 5.3. Efficiency Analysis of Aggregated Signature

Below, we compare and analyze the aggregate verification efficiency of the proposed aggregate signature scheme in our work with currently well-performing software and hardware implementation schemes from different perspectives. $P_{B}$ represents the bilinear pairing operation time on software, m represents the running time of a modular exponentiation on software, M represents the running time of a point multiplication on software, A represents the running time of a point addition on software, $M_{P}$ represents the running time of a point multiplication on the single-path FPGA in our work, $CoT_{P}$ represents the running time of a coordinate conversion operation on the single-path FPGA in our work, and n represents the number of signatures participating in the aggregation.

#### 5.3.1. Analysis of Non-Fault-Tolerant Aggregate Signature Verification Efficiency

First, let us assume that all signatures are valid and that the total number of signatures to be verified exceeds the 36 signatures required to construct a fault-tolerant set. Under this assumption, we can proceed with an analysis of the computational load associated with different aggregate signature schemes.

One such scheme, presented in [35], is a derivative of the fault-tolerant aggregate signature approach. To facilitate a meaningful comparison, the design of the fault-tolerant set in [35] has been aligned with the scheme proposed in our work. This unification allows for a direct comparison of the computational efficiency between the two methods, providing insights into their relative performance under similar conditions.

**Figure 7:**
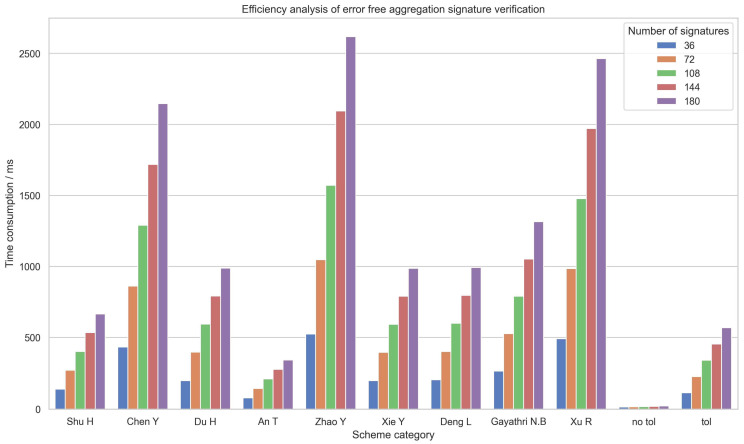
Efficiency analysis of error free aggregation signature verification.

As shown in Table 5, the time consumption formulas for individual signature verification and batch verification are provided. The efficiency of aggregate signatures is primarily determined by the efficiency of batch verification. Figure 7 displays a categorized bar chart of batch verification time consumption, where “no tol” represents the verification time in the non-fault-tolerant mode, and “tol” represents the consumption time in the fault-tolerant mode. From the figure, it can be seen that a significant difference in verification time emerges even with a small number of signatures. Therefore, we analyze the case with 36 signatures and no faults. The aggregate signature verification times from the literature are as follows: 139.532 ms in [36], 432.172 ms in [37], 199.013 ms in [38], 78.321 ms in [4], 523.692 ms in [35], 198.689 ms in [3], 205.142 ms in [39], 264.317 ms in [40], and 490.885 ms in [6]. The verification time in the fault-tolerant mode of our work is 113.459 ms, and it is 14.899 ms in the non-fault-tolerant mode. Compared to the original SM9 aggregate signature scheme, the verification time in the non-fault-tolerant mode of our work is reduced by 80.9%, and compared to other schemes, it is reduced by up to 97.1%. Although the time consumption in the fault-tolerant aggregate signature mode has increased compared to the original SM9 scheme, it has decreased by 78.3% compared to the fault-tolerant aggregate signature scheme in [35] and at least by 18.6% compared to other schemes. Additionally, it provides fault-tolerant aggregation functionality that other schemes do not possess.

#### 5.3.2. Analysis of Fault-Tolerant Aggregate Signature Verification Efficiency

Below, we analyze the fault-tolerant efficiency of the fault-tolerant aggregate signature, using an example where there are 36 signatures to be verified, including two erroneous signatures. First, we introduce the following theorem. Assume that set $\left(A_{1},A_{2},\ldots ,A_{n}\right)$ is a $uniform\left(k,n\right)$ construction of set A; then, for any r subset $A_{i1},A_{i2},\ldots ,A_{ir}\in \left\{A_{1},\ldots ,A_{n}\right\}\left(1\leq r\leq k-1\right)$, we have Equations (19) and (20), where $m=\left(\begin{array}{c} n\\ k-1 \end{array}\right)$.

(19) $$\left|\overset{r}{\underset{j=1}{\cup }}A_{ij}\right|=m-\left(\begin{array}{c} n-r\\ k-r-1 \end{array}\right)$$

(20) $$\left|\overset{r}{\underset{j=1}{\cap }}A_{ij}\right|=m-\left(\begin{array}{c} n\\ k-1 \end{array}\right)+\left(\begin{array}{c} n-r\\ k-1 \end{array}\right)$$

Similarly, the size of each subset $A_{i}$ is $\left(\begin{array}{c} n-1\\ k-1 \end{array}\right),1\leq i\leq n$. Specifically, when r takes the value of n−k+1, the value of Equation (20) is 1.

**Figure 8:**
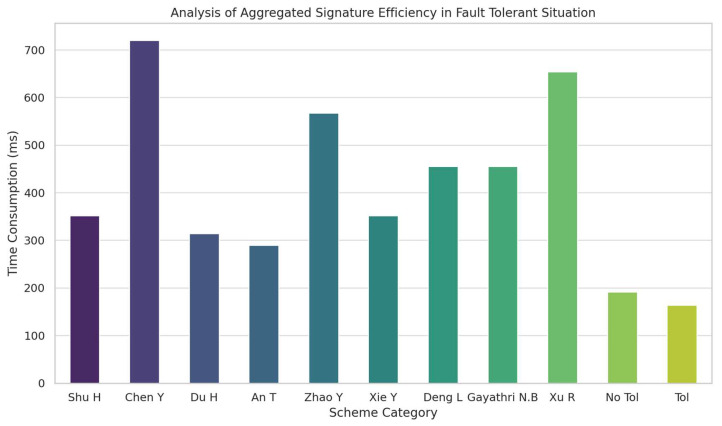
Efficiency analysis of fault-tolerant aggregate signature.

In our work, we construct a uniform(8,9) fault-tolerant aggregate signature set $\left\{T_{1},\ldots ,T_{9}\right\}$. Each $T_{i}\left(1\leq i\leq 9\right)$ has a size of $\left|T_{i}\right|=\left(\begin{array}{c} 9-1\\ 8-1 \end{array}\right)=8$. Once we have constructed the fault-tolerant set, for convenience of representation, we denote it using $\varepsilon_{1},\varepsilon_{2},\ldots \varepsilon_{9}$ to represent the corresponding $\left\{T_{1},\ldots ,T_{9}\right\}$. Additionally, we can observe that each individual signature appears twice in different $T_{i},T_{j}$. Given that $16\left|T_{i}\right|/36=2$, we can infer $\left|\cap_{j=1}^{2}A_{ij}\right|=\left(\begin{array}{c} 9-2\\ 8-1 \end{array}\right)=1$ from Equation (20) that the number of single signatures in the intersection of any two subsets is 1. Therefore, we can conclude that the two erroneous signatures will appear in three or four different $T_{i}$ subsets. In our analysis, we consider the worst-case scenario where the two erroneous signatures appear in four different $T_{i}$ subsets. Thus, only five subsets $\varepsilon_{i1},\varepsilon_{i2},\ldots \varepsilon_{i5}$ pass verification. Consequently, the number of correct signatures passing verification can be calculated using Equation (19), yielding $36-\left(\begin{array}{c} 9-5\\ 8-5-1 \end{array}\right)=30$. Therefore, a total of 30 signatures pass the verification. In error handling of aggregate signature, the method of independent verification after error is generally adopted. Below, we provide a brief explanation of the number of individual verifications required for non-fault-tolerant aggregate signatures. When two erroneous signatures are mixed into 36 signatures, assuming the probability of encountering the first erroneous signature at position $\left(0,35\right]$ is uniform, we denote its position as x. The probability of the second erroneous signature being at position $\left[x+1,36\right]$ is $1/\left(36-i\right)$. Therefore, the expected number of verifications is approximately 21. In contrast, the maximum number of verifications required for fault-tolerant aggregate signatures is six. Hence, the fault-tolerant aggregate signature scheme demonstrates better performance in the presence of erroneous signatures. Figure 8 presents the efficiency analysis of fault-tolerant aggregate signatures, where “no tol” represents the verification time in the non-fault-tolerant mode, and “tol” represents the consumption time in the fault-tolerant mode. Below, we provide more specific data: in this scenario, the time to verify and identify all erroneous signatures is 351.611 ms for [36], 720.25 ms for [37], 313.862 ms for [38], 289.833 ms for [4], 567.39 ms for [35], 351.632 ms for [3], 455.315 ms for [39], 455.354 ms for [40], and 653.782 ms for [6]. The non-fault-tolerant aggregate mode takes 191.206 ms, while the fault-tolerant aggregate mode takes 163.97 ms. In the current scenario, analyzing the time taken by each scheme, our proposed scheme, whether in fault-tolerant or non-fault-tolerant aggregate mode, significantly outperforms other non-fault-tolerant schemes. Even compared to the fault-tolerant scheme in [35], our scheme’s time consumption is only 28.9% of theirs. In summary, our designed scheme shows a substantial advantage in both fault-tolerant and fault-free scenarios.

## 6. Conclusions

Considering the complex network environment and high-performance requirements of vehicular networks, we design an efficient aggregate signature algorithm with two modes based on the SM9 aggregate signature algorithm, FPGA acceleration technology, and the *uniform*(*k*,*n*) theory. The security of the proposed algorithm is also analyzed. For the numerous elliptic curve point multiplication operations involved, a parallel point multiplication architecture based on FPGA is designed. Single-point multiplication uses the Montgomery point algorithm, and the data flow of the point addition and point double modules is optimized. Key modules for modular addition/subtraction, multiplication, and inversion are designed using combined modular addition/subtraction, KOA and Barret algorithms, and the binary modular inversion algorithm, respectively. The parallel point multiplication architecture is applied to the SM9 efficient aggregate signature scheme, and its effectiveness is verified through simulation and on-board experiments. A comprehensive analysis of the proposed scheme’s performance is also conducted. The highly parallel elliptic curve point multiplication acceleration module designed on the FPGA platform is applied to both non-fault-tolerant and fault-tolerant modes, demonstrating good performance in the low-latency and complex network environment scenarios of vehicular networks. Compared to similar schemes, the proposed scheme not only achieves higher operational efficiency but also incorporates fault-tolerant features.

The next step could explore the hardware security design of the SM9 parallel point multiplication architecture to prevent side-channel attacks. This involves ensuring high performance while preventing the leakage of computational information. Additionally, developing new fault-tolerance theories to further optimize the construction speed of fault-tolerant sets can be investigated.

## Figures and Tables

**Table 1:** Notations.

Notation	Descriptions
$P_{1},P_{2}$	Two different generators in the system
ks	System master private key
kpb	System master public key, $kpb=k\cdot P_{2}\left(k\in Z_{n-1}^{*}\right)$
N	The order of the elliptic curve group in SM9
$H_{1}\left(Z,n\right),H_{2}\left(Z,n\right)$	One-way hash function, which takes a bit string Z and an integer n as input, outputs h∈[1,n−1]
$ID_{i}$	The real identity of vehicle i
hid	Private key generation identifier
$VID_{i}$	Pseudonym assigned to vehicle i, denoted as $VID_{i}=H_{1}\left(ID_{i}||hid,N\right)$
$s_{i}$	Private key of vehicle i, $s_{i}=k\cdot {\left(VID_{i}+k\right)}^{-1}\cdot P_{1}$
$Q_{i}$	Public key of vehicle i, $Q_{i}=VID_{i}\cdot P_{2}+kpb$

**Table 2:** Point addition data flow.

Number	Operation	Computation
1	$T_{1}=X_{1}X_{2}$	$X_{1}X_{2}$
2	$z_{1}z_{2}=Z_{1}Z_{2}$	$Z_{1}Z_{2}$
3	$T_{2}=a\left(z_{1}z_{2}\right)$	$aZ_{1}Z_{2}$
4	$T_{1}=T_{1}-T_{2}$	$X_{1}X_{2}-aZ_{1}Z_{2}$
5	$T_{1}={\left(T_{1}\right)}^{2}$	${\left(X_{1}X_{2}-aZ_{1}Z_{2}\right)}^{2}$
6	$x_{1}z_{2}=X_{1}Z_{2}$	$X_{1}Z_{2}$
7	$x_{2}z_{1}=X_{2}Z_{1}$	$X_{2}Z_{1}$
8	$T_{2}=x_{1}z_{2}+x_{2}z_{1}$	$X_{1}Z_{2}+X_{2}Z_{1}$
9	$T_{2}=bT_{2}$	$b\left(X_{1}Z_{2}+X_{2}Z_{1}\right)$
10	$T_{2}=T_{2}\left(z_{1}z_{2}\right)$	$b\left(X_{1}Z_{2}+X_{2}Z_{1}\right)Z_{1}Z_{2}$
11	$T_{2}=T_{2}+T_{2}$	$2b\left(X_{1}Z_{2}+X_{2}Z_{1}\right)Z_{1}Z_{2}$
12	$T_{2}=T_{2}+T_{2}$	$4b\left(X_{1}Z_{2}+X_{2}Z_{1}\right)Z_{1}Z_{2}$
13	$X_{3}=T_{1}-T_{2}$	$\begin{array}{c} {\left(X_{1}X_{2}-aZ_{1}Z_{2}\right)}^{2}-\\ 4b\left(X_{1}Z_{2}+X_{2}Z_{1}\right)Z_{1}Z_{2} \end{array}$
14	$T_{1}=\left(x_{1}z_{2}\right)-\left(x_{2}z_{1}\right)$	$X_{1}Z_{2}-X_{2}Z_{1}$
15	$T_{1}={\left(T_{1}\right)}^{2}$	${\left(X_{1}Z_{2}-X_{2}Z_{1}\right)}^{2}$
16	$Z_{3}=x_{G}T_{1}$	$x_{G}{\left(X_{1}Z_{2}-X_{2}Z_{1}\right)}^{2}$

**Table 3:** Point double data flow.

Number	Operation	Computation
1	$x_{1}\_s={\left(X_{1}\right)}^{2}$	${\left(X_{1}\right)}^{2}$
2	$z_{1}\_s={\left(Z_{1}\right)}^{2}$	${\left(Z_{1}\right)}^{2}$
3	$az_{1}\_s=a\left(z_{1}\_s\right)$	$a{\left(Z_{1}\right)}^{2}$
4	$T_{1}=x_{1}\_s-az_{1}\_s$	${\left(X_{1}\right)}^{2}-a{\left(Z_{1}\right)}^{2}$
5	$T_{1}={\left(T_{1}\right)}^{2}$	${\left({\left(X_{1}\right)}^{2}-a{\left(Z_{1}\right)}^{2}\right)}^{2}$
6	$x_{1}z_{2}=X_{1}Z_{2}$	$X_{1}Z_{2}$
7	$\left({\left(X_{1}\right)}^{2}-a{\left(Z_{1}\right)}^{2}\right)X_{1}Z_{2}$	$b{\left(Z_{1}\right)}^{2}$
8	$T_{2}=\left(x_{1}z_{1}\right)\left(bz_{1}\_s\right)$	$bX_{1}{\left(Z_{1}\right)}^{3}$
9	$T_{2}=T_{2}+T_{2}$	$2bX_{1}{\left(Z_{1}\right)}^{3}$
10	$T_{2}=T_{2}+T_{2}$	$4bX_{1}{\left(Z_{1}\right)}^{3}$
11	$T_{2}=T_{2}+T_{2}$	$8bX_{1}{\left(Z_{1}\right)}^{3}$
12	$X_{3}=T_{1}-T_{2}$	${\left({\left(X_{1}\right)}^{2}-a{\left(Z_{1}\right)}^{2}\right)}^{2}-8bX_{1}{\left(Z_{1}\right)}^{3}$
13	$T_{1}=\left(x_{1}\_s\right)+\left(az_{1}\_s\right)$	${\left(X_{1}\right)}^{2}+a{\left(Z_{1}\right)}^{2}$
14	$T_{1}=T_{1}\left(x_{1}z_{1}\right)$	$\left({\left(X_{1}\right)}^{2}+a{\left(Z_{1}\right)}^{2}\right)X_{1}Z_{1}$
15	$T_{2}=\left(bz_{1}\_s\right)\left(z_{1}\_s\right)$	$b{\left(Z_{1}\right)}^{4}$
16	$T_{1}=T_{1}+T_{2}$	$\left({\left(X_{1}\right)}^{2}+a{\left(Z_{1}\right)}^{2}\right)X_{1}Z_{1}+b{\left(Z_{1}\right)}^{4}$
17	$T_{2}=T_{1}+T_{1}$	$2Z_{1}\left(X_{1}^{3}+aX_{1}Z_{1}^{2}+bZ_{1}^{3}\right)$
18	$Z_{2}=T_{2}+T_{2}$	$4Z_{1}\left(X_{1}^{3}+aX_{1}Z_{1}^{2}+bZ_{1}^{3}\right)$

**Table 4:** Resource utilization and performance of FPGA modules.

Module	Control Mode	Operation Mode	LUT	FF	DSP	Frequency/MHZ	Clock Cycles
modular inversion	State Machine	Serial	3951	1568	0	27	544
modular addition and subtraction units	Pure Combinational Logic	Parallel	2918	0	0	27	1
modular multiplication	State Machine	Serial	4581	2054	144	27	6
point addition	State Machine	Parallel	8137	5168	144	27	102
point double	State Machine	Parallel	8003	5664	144	27	102
point multiplication	State Machine	Parallel	20,321	15,505	288	27	26,014
coordinate conversion	State Machine	Serial	18,091	7618	288	27	1453
slave state machine	State Machine	Parallel	4181	4673	0	27	-
master control state machine	State Machine	Parallel	10,012	23,471	0	50	-

**Table 5:** Comparison of individual and batch verification costs in different aggregate signature schemes.

Module	Individual Verification	Batch Verification
Shu, H. [36]	$2P_{B}+M+3A$	$2P_{B}+2nM+2\left(n+1\right)A$
Chen, Y. [37]	$2P_{B}+3M+2A$	$2nP_{B}+\left(2n+1\right)M+2nA$
Du, H. [38]	3M+3A	$\left(3n+1\right)M+\left(4n-1\right)A$
An, T. [4]	$2P_{B}+M$	$3P_{B}+nM+2\left(n-1\right)A$
Zhao, Y. [35]	4M+3A	$8nM+\left(4n-4\right)A$
Xie, Y. [3]	4M+3A	$\left(3n+1\right)M+\left(3n-1\right)A$
Deng, L. [39]	$2P_{B}+2M+3A$	$2P_{B}+3nM+3nA$
Gayathri, N.B. [40]	5M+3A	$\left(4n+1\right)M+\left(4n-1\right)A$
Xu, R. [6]	$P_{B}+2M$	$\left(2n-1\right)P_{B}+\left(3n+1\right)M$
Non-fault-tolerant mode	$2P_{B}+M$	$3P_{B}+\left\lceil \frac{n}{72}\right\rceil M_{P}+3\left(n-1\right)A+\left\lceil \frac{n}{72}\right\rceil CoT_{P}$
Fault-tolerant mode	$2P_{B}+M$	$3\left\lceil \frac{n}{4}\right\rceil P_{B}+\left\lceil \frac{n}{36}\right\rceil M_{P}+3\left(n-1\right)A+\left\lceil \frac{n}{36}\right\rceil CoT_{P}$

## Data Availability

Data are contained within the article.
